# Evaluation of Cellular Uptake and Removal of Chlorpropham in the Treatment of *Dunaliella salina* for Phytoene Production

**DOI:** 10.3390/md20060367

**Published:** 2022-05-30

**Authors:** Laura Mazzucchi, Yanan Xu, Patricia J. Harvey

**Affiliations:** School of Science, Faculty of Engineering and Science, University of Greenwich, Central Avenue, Chatham Maritime, Kent ME4 4TB, UK; l.mazzucchi@greenwich.ac.uk (L.M.); y.xu@greenwich.ac.uk (Y.X.)

**Keywords:** *Dunaliella salina*, chlorpropham, herbicide, phytoene, carotenoids

## Abstract

Chlorpropham is a carbamate herbicide that inhibits cell division and has been widely used as a potato sprout suppressant. Recently we showed that the microalga *Dunaliella salina* treated with chlorpropham massively accumulated the colourless carotenoids phytoene and phytofluene. Phytoene and phytofluene are valued for their antioxidant, UV-absorption and skin protectant properties; however, they are present in very low quantities in nature. The low toxicity herbicide chlorpropham seems a promising catalyst to produce phytoene in large quantities from CO_2_ and solar energy with *D. salina*. This study explored chlorpropham uptake by the algal cells, the formation of potential intermediate metabolites, and the removal of residual chlorpropham from harvested *D. salina* biomass. Algal biomass rapidly concentrated chlorpropham from culture media. However, washing the harvested biomass with fresh culture medium twice and five times removed ~83 and ~97% of the chlorpropham from the biomass, respectively, and retained algal cell integrity. Furthermore, chloroaniline, a common metabolite of chlorpropham degradation, was not detected in chlorpropham-treated cultures, which were monitored every two days for thirty days. Cells treated with chlorpropham for either 10 min or 24 h continued to over-accumulate phytoene after resuspension in an herbicide-free medium. These data imply that whilst *Dunaliella* cells do not possess the intracellular capacity to degrade chlorpropham to chloroaniline, the effect of chlorpropham is irreversible on cell nuclear division and hence on carotenoid metabolism.

## 1. Introduction

Chlorpropham (CIPC), isopropyl 3-chlorocarbanilate (IUPAC), is a carbamate herbicide and is very widely used worldwide as a general plant growth regulator to control sprouting and as a herbicide against target weeds [1,2]. Recently, Xu and Harvey [3] showed that the addition of micromolar quantities of chlorpropham to cultures of the microalga, *Dunaliella salina,* inhibited cell division and resulted in the massive overaccumulation of the colourless carotenoids, phytoene and phytofluene. *D. salina* is a halotolerant marine microalga and well known for producing high quantities of β-carotene [4]. Phytoene and phytofluene are precursors of β-carotene and are naturally found in a limited amount in the alga. Chlorpropham was proposed to disrupt synchronised control between nuclear and chloroplast events in cell division, which would normally be associated with carotenogenesis and β-carotene accumulation [4].

Phytoene and phytofluene are similar to β-carotene in being comprised of a C40 backbone of isoprenoid units, which confer antioxidant and anti-inflammatory properties [5]. Unlike β-carotene, however, the absorbance maxima for phytoene and phytoene lie within the UV wavelength range (280–350 nm). Phytoene and phytoene are consequently sought after as UV-protective ingredients in skin protection products [6,7]. Both compounds are found naturally in various stereoisomeric forms, which differ in their physicochemical properties and shape. In simulated gastro-intestinal studies, *cis*-isomers of phytoene and phytofluene from different fruit juices presented with a higher bioaccessibility than their *trans*-isomer counterparts, suggesting that they may have a higher therapeutical value [8]. The isomers from *D. salina* were recently comprehensively characterised and quantified after treatment of *D. salina* cultures with chlorpropham [7]. *Cis-* forms of the isomers are the predominant form in *D. salina.* However, phytoene and phytofluene normally are only found in a limited amount in *D. salina.* This has, until now, limited the potential for industrial-scale developments using this alga [9].

To date, chlorpropham is known to inhibit the process of mitosis in plants and algae by interfering with the spindle microtubule organising centre, causing abnormal or complete suppression of microtubule synthesis and organisation. However, its specific site of action is not yet known [10]. Sterling [11] showed that lipophilic and neutral herbicides, including chlorpropham, could penetrate the cell membrane of some lower and several higher plants by passive diffusion; the kinetics studies of the uptake and efflux of different neutral pesticides have been cited. Little is known about the cellular uptake and accumulation of chlorpropham by microalgae and specifically *D. salina*. Likewise, the biotransformation of chlorpropham has been investigated in higher plants and mainly in potatoes due to its extensive use on this crop. In studies performed with potatoes, chlorpropham was shown to be decarboxylated to 3-chloroaniline (3-CA) [12], which is an organochlorine compound listed on the European Community priority pollutant Circular No 90–55 (1990) [13], and classified as highly toxic for the environment and humans. Studies on the metabolism of chlorpropham in algae are limited. To our knowledge, only John et al. [14] investigated its degradation by a selection of green algae (such as *Chlorella pyrenoidosa* and *Chlamydomonas, Ulothrix fimbriata*) and blue green algae (*Anacistis nidulans*). Using colorimetric methods for detection of 3-CA, they observed that only *A. nidulans* transformed chlorpropham to 3-CA.

The aim of the present work was to gain insights into the interaction between the herbicide chlorpropham and *D. salina* cells by studying its uptake and possible metabolism to 3-CA in cell cultures of *D. salina*. The objectives involved the detection of chlorpropham and its metabolites in the algal biomass, the study of its concentration over time and its removal from the biomass.

## 2. Results

### 2.1. Determination of Chlorpropham and Its Metabolites in Algal Biomass and Extracts

#### 2.1.1. HPLC Detection of Chlorpropham and 3-Chloroaniline

**Figure 1:**
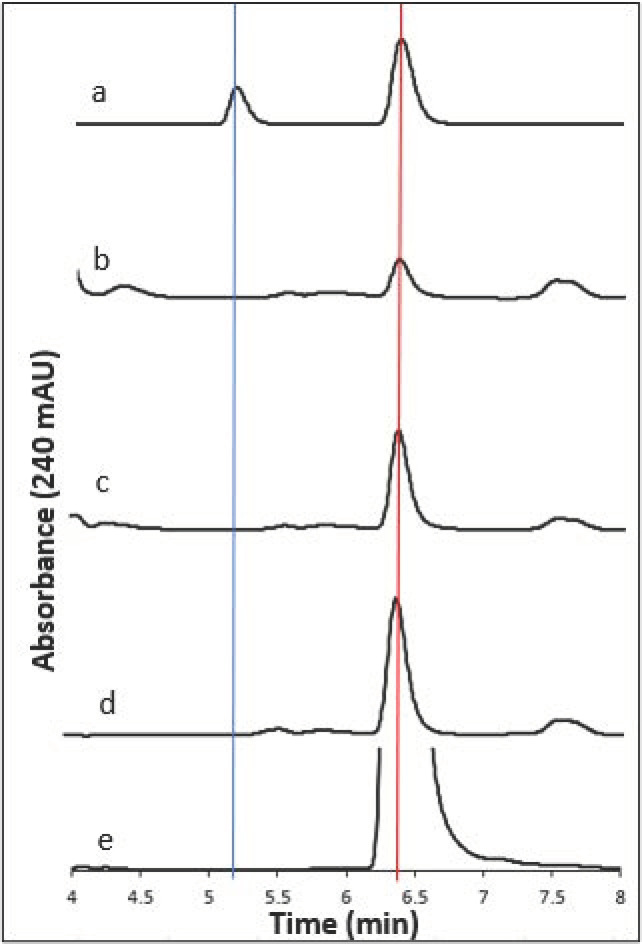
HPLC chromatograms of (a) chlorpropham (7.5 µg/mL) and 3-chloroaniline (1 µg/mL) standard spiked in *D. salina*, and extracts from *D. salina* treated with (b) 20 µM, (c) 50 µM, (d) 200 µM and (e) 1 mM chlorpropham for one month; red line (RT = 6.39 min) highlights the peaks corresponding to chlorpropham; blue line (RT = 5.24 min) indicates peak of 3-chloroaniline. n.d. not detected.

Harvested biomass from *D. salina* cultures treated with 20 µM–1 mM chlorpropham over 30 days was extracted with methanol for chlorpropham and 3-chloroaniline, and the extracts were analysed by HPLC at an interval of 2 days for 30 days. HPLC chromatograms of the extracts revealed the presence of chlorpropham in the treated biomass, based on the spectral properties of pure standard and its retention time (5 min, see Figure 1), but 3-chloroaniline could not be detected (detection limit 1 ng/mL). The recovery efficiency of 3-chloroaniline from *D. salina* biomass was assessed with the 3-chloroaniline standard using methanol and determined to be 91%. These data suggested that chlorpropham was not metabolised to 3-chloroaniline by *D. salina* cells.

#### 2.1.2. Determination of Chlorpropham and Phytoene in *D. salina* Biomass

**Figure 2:**
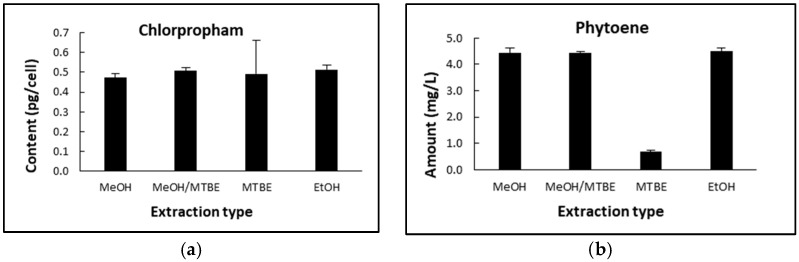
(**a**) Chlorpropham content with different extraction solvents; (**b**) phytoene yield with different extraction solvents.

Figure 2 shows the quantification of chlorpropham and phytoene in *D. salina* extracts from unwashed biomass, which was extracted with either MeOH, MeOH/MTBE, MTBE or EtOH. As shown in Figure 2a, the chlorpropham contents in the extracts with different solvents were not significantly different (*p* > 0.05, Anova), whereas the phytoene contents of the extracts (Figure 2b) were significantly different (*p* < 0.001, Anova); extraction with either EtOH, MeOH or MeOH/MTBE yielded similar levels of phytoene (*p* > 0.05), whilst MTBE gave the lowest yield of phytoene. In all subsequent experiments, MeOH was therefore used to assess the contents of phytoene and chlorpropham in harvested biomass.

### 2.2. Cellular Uptake and Accumulation of Chlorpropham during D. salina Cultivation

#### 2.2.1. Chlorpropham Uptake over Time

**Figure 3:**
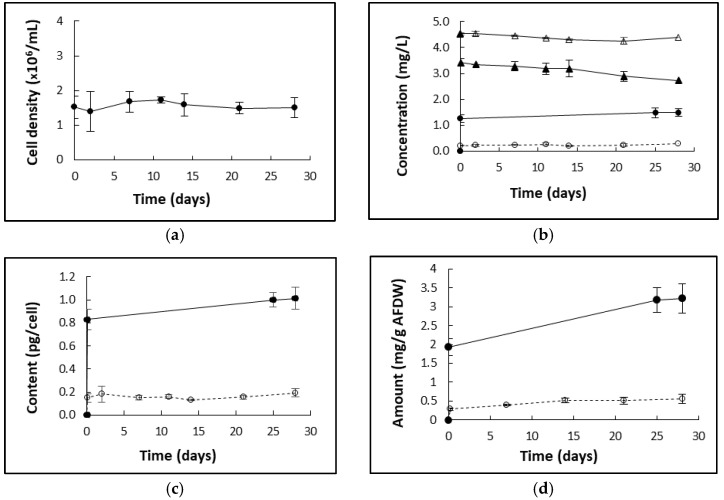
(**a**) The cell density of the *D. salina* cultures treated with 20 µM chlorpropham; day 0 represents the start of the treatment. (**b**) Changes in chlorpropham amount (mg/L culture) over time in different fractions of *D. salina* cultures treated with 20 µM chlorpropham; unwashed biomass after centrifugation (circle, full line), biomass washed twice with fresh culture medium (broken line), supernatant from centrifugation (full triangle) and chlorpropham solution control that was not inoculated (open triangle); (**c**) cellular content of chlorpropham in unwashed biomass of *D. salina* cultures treated with 20 µM herbicide (full line) and washed twice (broken line); (**d**) chlorpropham (mg/g AFDW) in *D. salina* total biomass treated with 20 µM herbicide when cells were not washed (full line) and washed twice (broken line).

The kinetics of binding and uptake of chlorpropham by *D. salina* cells is shown in Figure 3, which displays chlorpropham amounts in the harvested biomass and culture medium over a period of 30 days. After 5 min following the addition of chlorpropham to cultures, unwashed biomass contained 1.92 ± 0.23 mg chlorpropham/g biomass (AFDW). Over the next 28 days, there was no further significant increase in cell density (*p* > 0.05). However, the amount of chlorpropham continued to associate with the biomass at a linear rate of 0.04 mg/day and, after 28 days, reached 3.22 mg/g AFDW. In concert, a corresponding significant decrease (*p* < 0.05) of chlorpropham amount in the culture medium between day 0 and day 28 (from 4.31 to 2.59 mg/L) was recorded (see Figure 3). Chlorpropham solution in uninoculated control had no significant change (*p* > 0.05) in chlorpropham concentration over the same period.

On the other hand, the amount of chlorpropham associated with washed biomass compared to unwashed never reached more than the initial concentration of 0.2 pg/cell throughout the 28-day test period, indicating that only a small fraction of the original amount of chlorpropham added to cultures might be needed to solicit phytoene accumulation (Figure 3c,d).

#### 2.2.2. Effect of Chlorpropham Concentration

**Figure 4:**
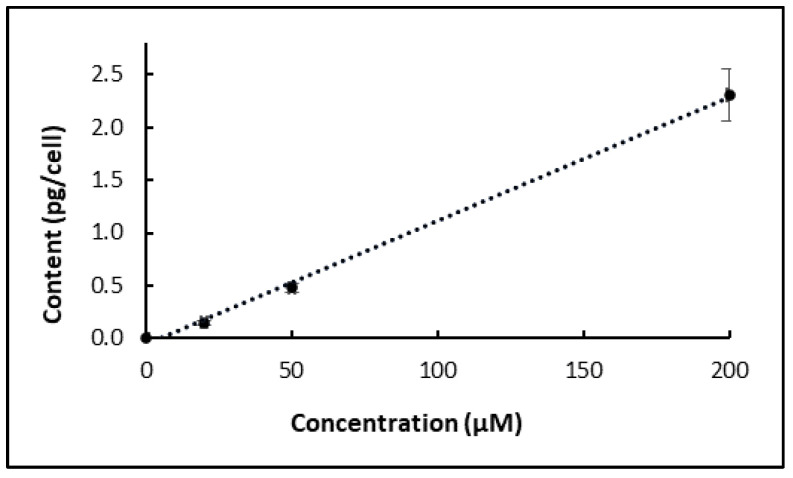
Correlation between the concentration of chlorpropham added to *D. salina* cultures and the chlorpropham content in *D. salina* cells (the concentration was measured after 24 h of treatment).

Different concentrations of chlorpropham were added to *D. salina* cultures, and the amount of chlorpropham taken up by the cells increased linearly with the increasing concentration of the chlorpropham added into the cultures (Figure 4). In 50 µM chlorpropham-treated cultures, the amount of chlorpropham in the cells was three times higher than that in 20 µM chlorpropham-treated cells. Additionally, in 200 µM chlorpropham-treated cultures, the amount was 15 times higher than that in 20 µM treated cultures.

#### 2.2.3. Effect of Cell Density

**Figure 5:**
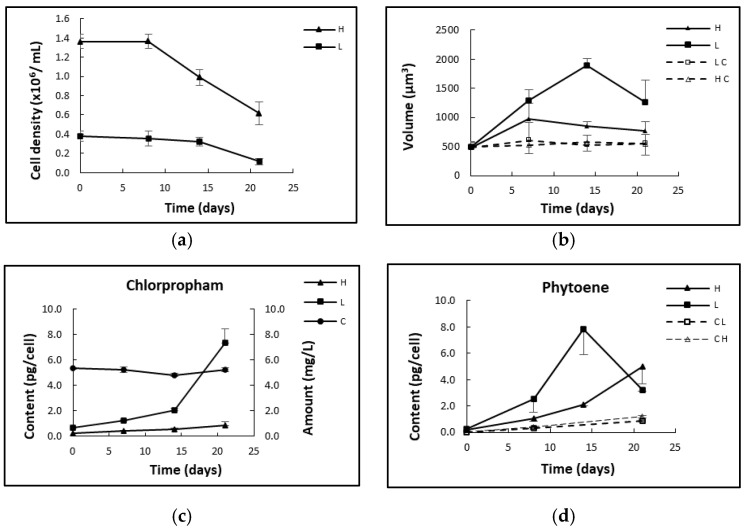
(**a**) Cell densities of the cultures treated with 20 µM chlorpropham. (**b**) Cell volume measurements of cultures treated at the cell densities of 1.36 × 10^6^ cells/mL (H) and 0.4 × 10^4^ cells/mL (L); (**c**) Chlorpropham content (pg/cell) in cultures at different cell densities (H and L in the figure) and in not inoculated control cultures; (**d**) Phytoene content in the biomass of low- and high-density-treated cultures or untreated controls (CH/CL).

**Figure A1:**
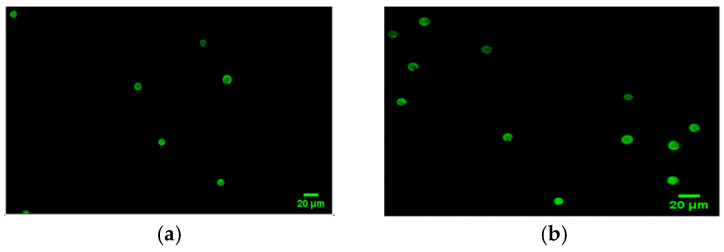

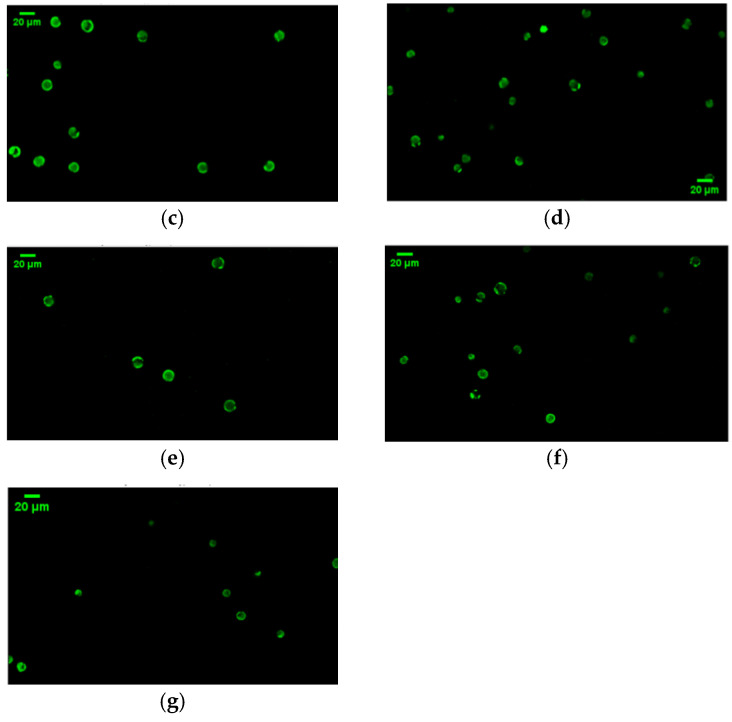
Confocal microscopy images of cultures treated with 20 μM of chlorpropham (**a**) low density, time 0; (**b**) high density, time 0; (**c**) low density, time 7; (**d**) high density, time 7; (**e**) low density, time 15; (**f**) high density, time 15; (**g**) high density, time 22.

Two sets of cultures with different cell densities (1.36 × 10^6^/mL and 0.4 × 10^6^/mL) were treated with 20 µM chlorpropham to study the effect of different cell densities on chlorpropham accumulation. The data in Figure 5 show that cell volume overall increased (significantly) from day 0 onwards in all the cultures (*p* < 0.05), in line with the progressive increase in the phytoene content and chlorpropham amount in the cells. See also Figure A1, Appendix A. The increasing cellular content of phytoene over time (Figure 5d) may contribute to cell swelling reflected in a slightly increased rate of accumulation of chlorpropham during the treatment period (Figure 5c). The volume increase was significantly higher in cultures of lower density. Notably, from day 22 of chlorpropham treatment, cells of cultures with low density became pale and irregular in shape, and cultures turned light grey, intracellular phytoene significantly decreased (*p* < 0.05) and chlorpropham associated with this biomass sharply increased (*p* > 0.05) between day 15 and 22.

### 2.3. Process Optimisation to Reduce the Concentration of Chlorpropham Loosely-Associated with Algal Biomass

#### 2.3.1. Different Numbers of Washing Cycles

**Figure 6:**
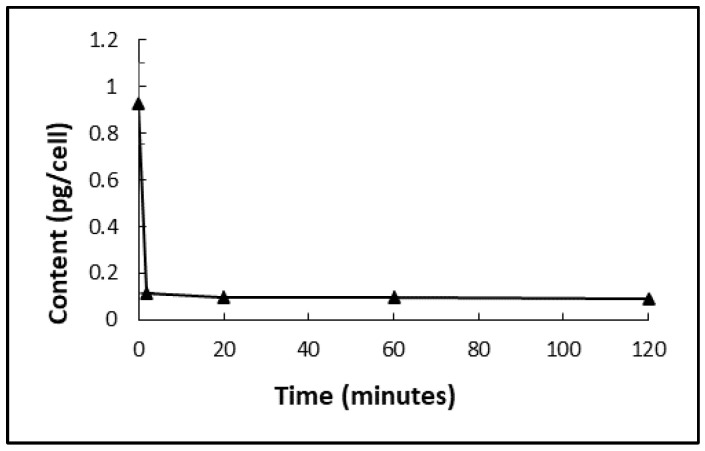
Loss of cellular chlorpropham amount in the harvested *D. salina* cells treated with 20 µM of chlorpropham. Unwashed cell pellets were obtained from 30 mL of treated cultures and then transferred to 60 mL of fresh herbicide-free medium.

Harvested *D. salina* biomass was washed with a fresh culture medium, and the effectiveness was assessed so that the amount of chlorpropham associated with the biomass and thereafter the phytoene extracts could be minimised. Treated *D. salina* cells were centrifuged, and pellets were transferred into a fresh herbicide-free medium. The change in the chlorpropham content in the harvested biomass was monitored, as shown in Figure 6. The results show that almost 90% of the chlorpropham associated with unwashed *D. salina* cells was released into the washing medium after two minutes of transferring the treated biomass into the fresh medium, indicating the release of chlorpropham from the cells to the fresh medium occurred very rapidly.

**Figure 7:**
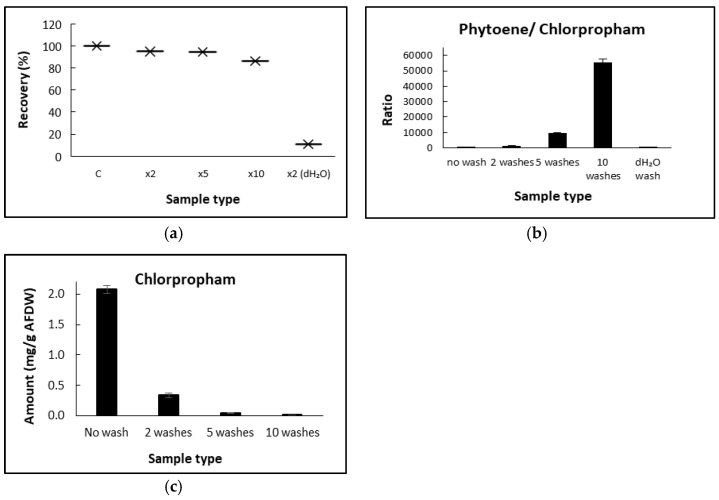
(**a**) Mean recovery (%) of phytoene from biomass after washing with fresh medium or water for different times. (**b**) phytoene/chlorpropham ratios when cells are not washed (1), washed twice (2), washed 5 times (3), 10 times (4) and washed with water (5). (**c**) Amount of chlorpropham in the final *D. salina* biomass (mg/g).

Table 1 and Figure 7 show chlorpropham removal and phytoene recovery from harvested biomass with different numbers of washing cycles. A significant difference between the content of chlorpropham derived from washed and unwashed biomass was found (*p* < 0.05). Chlorpropham contents in the biomass washed either 2, 5 or 10 times were 6.2, 45.5 and 307 times lower, respectively, than the unwashed biomass. More than 99% of chlorpropham in the harvested biomass was removed after washing with a fresh medium 10 times. Given that the harvested pellets had a volume of ~50 µL in each test and that the concentration of chlorpropham in the culture was 20 µM, the calculated herbicide carried over by water in the pellet volume was ~0.21 µg. This value was 85 times and 18 times lower in respect of the total amount of chlorpropham in both unwashed (18 µg) and washed biomass (3.75 µg) extracted from the pellet, suggesting that the biomass had concentrated chlorpropham from the extracellular medium.

Since *D. salina* cells lack a cell wall and are therefore relatively easily ruptured, quantification of phytoene in harvested biomass served as an internal marker and the ratio of chlorpropham:phytoene was recorded. The results show that the total cells that remained intact after washing 2, 5 or 10 times with fresh medium were 98.5%, 94% and 61.1%, respectively, in comparison to the biomass harvested without washing. Additionally, the recovery of phytoene was also investigated from samples when the volume ratio of washing solution:harvested cultures was 2:1 (Figure 7a). More than 90% of phytoene was recovered when *D. salina* biomass was washed less or equal to 5 times, and more than 80% recovered when biomass was washed 10 times.

Washing *D. salina* cells with water caused a substantial loss of phytoene (90% of total phytoene). The ratio of phytoene/chlorpropham increased with increasing washing cycles (Figure 7b), but it remained small when cells were washed with water, which caused complete cell disruption. Washing with water ruptured cells as indicated both visually and by the low value obtained for the ratio of chlorpropham:phytoene. These results confirm that washing with a culture medium successfully removes chlorpropham from *D. salina* biomass without bursting the cells.

#### 2.3.2. Different Washing Volumes

Table 2 shows that the decrease in chlorpropham in harvested biomass was positively correlated to the increase in wash solution: harvest volume ratio. Chlorpropham amount in the biomass was 8.9 times lower than that recorded in unwashed biomass when the volume ratio of washing solution: harvest culture was 1:1; 17.8 times lower when the ratio was 2:1, and 71.6 times lower when the ratio was 4:1 (Table 2).

#### 2.3.3. Phytoene Production in Washed D. salina Cells after Chlorpropham Treatment

**Figure 8:**
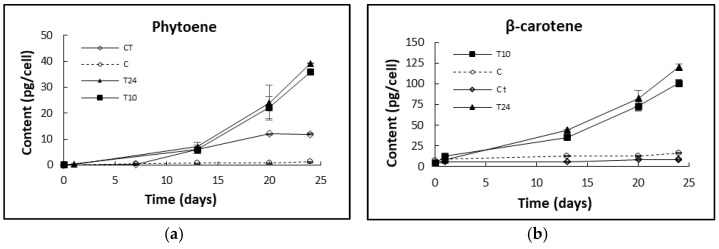
(**a)** Phytoene and (**b**) β-carotene content (pg/cell) in the different cultures (T10 = 10 min treatment; T24 = 24 h treatment; C = control; C t = control treated).

**Figure A2:**
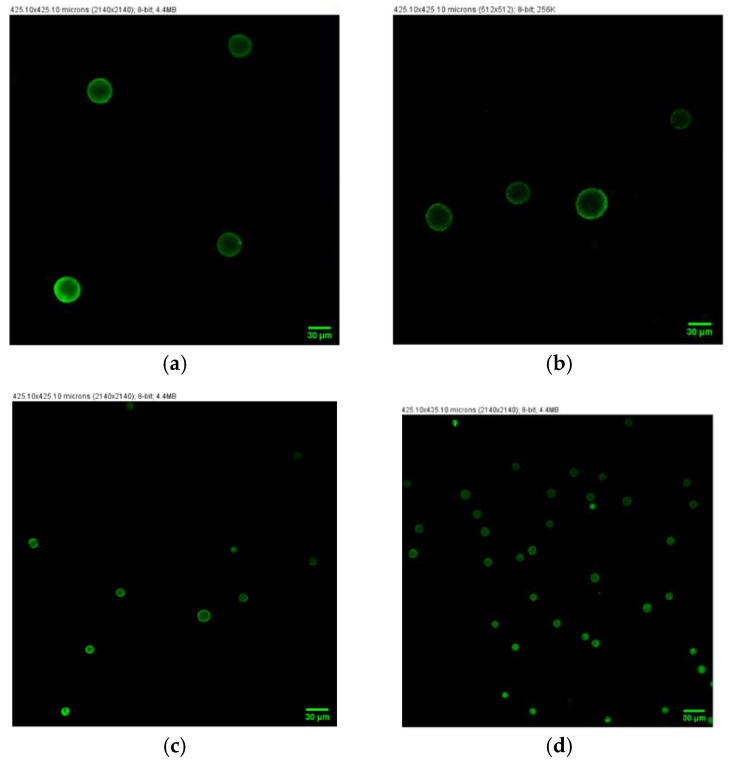
Confocal microscopy images of cultures treated with 20 µM of chlorpropham for (**a**) 10 min, (**b**) 24 h, (**c**) all the experimental period, and (**d**) not treated cultures at day 20 of the experiment.

*D. salina* cultures treated with chlorpropham for either 10 min or 24 h were harvested, and then pellets were resuspended in herbicide-free fresh medium to investigate the remained effects of chlorpropham on algal cells in terms of carotenoid accumulation (Figure 8; Figure A2, Appendix A). Cells harvested from both treated cultures continued to accumulate phytoene as well as β-carotene at significantly faster rates than in controlled, treated cultures. This is probably because the cells were diluted to lower densities in the fresh medium and gained access to higher light energy as well as higher nutrient levels. There is no significant difference between the cells treated for 10 min and those treated for 24 h (*p* > 0.05), confirming that the effects occurred in the cells rapidly within the first few minutes.

## 3. Discussion

Chlorpropham is a plant herbicide and a mitotic inhibitor which has been used extensively as a potato sprout suppressant. When *D. salina* cultures were treated with a micromolar concentration of chlorpropham, the two colourless carotenoid precursors, phytoene and phytofluene, massively accumulated [3]. The present work was undertaken to explore the interaction between chlorpropham and *D. salina* cells and the accumulation and removal of chlorpropham in the algal biomass.

*D. salina* biomass concentrated chlorpropham from the culture medium, but most could be removed by washing the biomass. A similar phenomenon has been previously reported using higher plant tissues [15]: in the study of the absorption of C-labelled herbicides washed for different cycles and time periods, herbicides such as fluorodifen (log K ow of 1.84 at 25 °C), which were concentrated by higher plant tissues, were removed after 10 min washing period. In the present study, the fresh culture medium was chosen as the washing solution to maintain the osmotic pressure and reduce the likelihood of cell rupture during washing since *D. salina* cells have no rigid cell wall [16]. Under these circumstances, the intracellular phytoene content remained within the cells.

Monitoring of the chlorpropham uptake by *D. salina* over time showed that the association of chlorpropham with the harvested biomass occurred within the first few minutes of treatment. Lipophilic herbicides have been shown to freely transfer across the cell membrane of cells via passive diffusion until the chemical equilibrium between the internal and external concentration is reached [11] and at a rate dependent on their lipophilicity. Chlorpropham is relatively lipophilic with a partition coefficient of octanol/water = 5.75 × 10^3^. *D. salina* cells, moreover, are bounded from the extracellular medium by a lipophilic membrane comprising a glycocalyx-like cell layer of varying thickness [16]. Although the precise site of action of chlorpropham is not clear, it is likely to be internal, as chlorpropham is known to disrupt mitosis by interfering with the spindle microtubule organising centres in lower and higher plants species [10,17] or by interacting with the microtubules directly [18]. In *D. salina* cultures, chlorpropham may be taken up by cells by internal passive diffusion through the cell membrane; this needs further exploration. In the present study, *D. salina* cells treated with chlorpropham for either 10 min or 24 h continued to accumulate phytoene even after their transfer into herbicide-free fresh medium and at the same rate as those cells that remained in chlorpropham-containing culture medium.

Chlorpropham accumulation during cell growth may parallel the increase in cell volume; increases in cell volume in cells treated with chlorpropham have been shown previously [19]. Our data show that the content of phytoene significantly accumulated over the period tested (21 days), and cell volumes of all treated cultures were observed to increase over time after the treatment while phytoene and phytofluene were accumulated, in accord also with [3]. Additionally, the sharp increase in volume in cultures of low cell number paralleled higher carotenoid accumulation, suggesting a correlation between *D. salina* cell number and the amount of chlorpropham added. Therefore, the physiological changes observed on day 21 of treatment in cultures with lower cell density may be linked to a higher toxicity effect of intracellular herbicide. It was shown previously that cell swelling occurred in cells grown once a specific level of chlorpropham was reached [20]. On the other hand, when cultures were treated for only 10 min or 24 h and resuspended in fresh medium, the effect of chlorpropham on the overaccumulation of phytoene was shown even after 24 days, proving the irreversibility of the herbicide after short exposure without showing cell toxicity caused by prolonged exposures to the herbicide.

The formation of the metabolite, 3-chloroaniline, has been a concern for chlorpropham applications. In this study, no 3-chloroaniline was detected in cultures treated with up to 1 mM herbicide with the method used, suggesting that *D. salina* might not be capable of breaking down chlorpropham. There is little information available regarding the degradation of chlorpropham in microalgal species. John et al. [14] investigated the presence of 3-chloroaniline in different microalgae and a cyanobacterium and showed that *Ulothrix fimbriata*, which belongs to the same phylum *Chlorophyta* as *D. salina*, did not produce 3-chloroaniline, in contrast to the blue-green alga *Anacystis nidulans*, which possessed the enzyme acylamidase. On the other hand, studies on potato tissues attributed the presence of 3-chloroaniline after chlorpropham treatment to the thermal degradation of chlorpropham during the fogging application [21], when extremely high temperatures (>300 °C) are used; high temperatures have been reported to trigger the degradation to 3-chloroaniline [22]. Additionally, 3-chloroanline may form during potato storage due to the activity of microorganisms capable of degrading the parent compound to this metabolite [23].

The present work aimed to elucidate details of the use of chlorpropham in *D. salina* cultivation for phytoene production. Chlorpropham added to cultures was concentrated in the biomass. The effects caused by chlorpropham on cell metabolism are irreversible. The constant increase in the chlorpropham content associated with the biomass over time could be linked to increased cell volume during the accumulation of the colourless carotenoids, phytoene and phytofluene. Washing repeatedly with fresh medium will remove most chlorpropham associated with harvested biomass, up to more than 99% of its initial amount. Phytoene and phytofluene are high valued compounds which are sought to have high beneficial health properties; however, their availability in nature is low. The use of chlorpropham with *D. salina* cultivation may represent a facile, low-cost method for producing large quantities of phytoene.

## 4. Materials and Methods

### 4.1. Alga Strain and Cultivation

*D. salina* strain DF15 (CCAP 19/41) was obtained from the Marine Biological Association, UK (MBA). Cultures were cultivated in 500 mL Modified Johnsons Medium [24] containing 1.5 M NaCl and 10 mM NaHCO_3_ in an illuminated incubator (Varicon Aqua, Worcester, UK) under white light of 500 µmol m^−2^ s^−1^ at 25 ± 2 °C. For the different kinetic studies of treatments with herbicides, triplicate sets of cultures were grown to mid-late log phase, and each set was treated with chlorpropham at different concentrations from 20 µM up to 1 mM. Flasks containing only fresh culture media added with the same amount of herbicide served as blank controls to monitor the natural degradation rate of chlorpropham over time without algal cells.

### 4.2. Standards and Solvents

Phytoene standard (LGC Limited, Teddington, UK), chlorpropham (PESTANAL, analytical standard) and 3-chloroaniline (99% purity) were purchased from Sigma-Aldrich (Merck KGaA, Darmstadt, Germany). Methanol (MeOH) and Methyl Tert Butyl Ether (MTBE), both HPLC grade, were purchased from Fischer Scientific UK Ltd. (Loughborough, Leicestershire, UK).

### 4.3. Extraction and Analysis of Phytoene

To determine the yield of phytoene and other carotenoids in chlorpropham-treated and control cultures of *D. salina* at different time periods, 5 to 10 mL of *D. salina* cultures were harvested at 3000× *g* for 5 min at 20 °C with an Eppendorf centrifuge 5810R (Eppendorf, UK). Carotenoids were extracted and analysed by HPLC as described in our previous work [7]: 10 mL of MeOH: MTBE (80:20) was added to the samples, which were first sonicated for 60 s and then vortexed for 20 s. Samples were clarified at the centrifuge, and the top solvent phase was collected. The extracts were filtered (0.20 µm filter) into amber HPLC vials before analysis. The carotenoid extracts were routinely flushed with nitrogen after extraction. To determine the kinetics for phytoene accumulation, *D. salina* cultures (cell density = 85.5 × 10^4^/mL) were treated with chlorpropham for either 10 min or for 24 h, and then aliquots of the cultures (30 mL) were transferred to fresh, herbicide-free, culture medium (60 mL), and the rate of phytoene accumulation (pg/cell) determined for 24 days. The phytoene yield in those cultures was compared to cultures not treated and cultures treated for all the 24 days analysis period. To determine the yield of phytoene, a calibration curve was generated for its quantification.

### 4.4. Extraction and Analysis of Chlorpropham and Its Potential Metabolites

#### 4.4.1. Sample Preparation

Analytical methods for chlorpropham and its metabolite 3-chloroaniline include high-performance liquid chromatography or gas chromatography. However, gas chromatography often requires the derivatisation of the compounds before analysis and, therefore, HPLC was selected as the main tool for chlorpropham and 3-CA analysis in this study. To monitor the amount of chlorpropham in *D. salina* cells on different days of treatment (day 0 to day 28), 10 to 15 mL cultures were harvested at 3000× *g* for 5 min at 20 °C. The extraction of chlorpropham from *D. salina* biomass was evaluated with the following solvent extracts: MeOH (100%), MTBE (100%), Ethanol (100%), MTBE/MeOH (20/80). 2.5 mL MeOH (100%) was added to the biomass, and the solvent suspension was sonicated for 20 s, vortexed for 20 s and centrifuged at 3,000× *g* for 5 min. The upper phase with the solvent was collected, filtered with 0.2 µm size filters into amber HPLC vials and analysed by HPLC.

To enhance the compatibility with the HPLC mobile phase, as a stronger sample phase solvent than the mobile phase may lead to inaccuracies in the results, a final sample phase consisting of 90% sample (in MeOH) and 10% of HPLC grade water was prepared. To analyse samples deriving from the algal supernatant, 5 mL of each collected supernatant was diluted with 15 mL of MeOH (HPLC grade) to reach a ratio (25:75, H_2_O/MeOH).

#### 4.4.2. HPLC Analysis of Chlorpropham and 3-Chloroaniline

The presence of chlorpropham and its metabolite, 3-chloroaniline, was evaluated with HPLC by matching the retention time and the UV-vis spectra features of authentic standards; these values are shown in Table 3. The instrument was equipped with Diode-Array Detection (HPLC-DAD; Agilent Technologies 1200 series, Agilent, Santa Clara, CA, USA), an online degasser and a quaternary pump system. Samples were separated using a C18 250 × 4.6 mm Waters Spherisorb 5 µm ODS2. MeOH/ dH_2_O mixtures with different ratios of water (from 0 to 10%) were tested as mobile phase solvents for analysis. The method parameters chosen were as follows: the column temperature was set at 20 °C, and the gradient solvent system was MeOH/ dH_2_O (90/10) (A) running at 100% A for the first 8 min at a flow rate of 0.6 mL/min, then running at 100% MeOH (B) for one minute, before adjusting to 80% B and 20% C (C = 100% MTBE) for the next 20 min at a flow rate of 1.0 mL/min (to elute the carotenoids) before going back to the initial conditions at 30 min. The total run time was 45 min. The absorbance at different wavelengths (210, 240, 250, 282, 355, 450, 480 nm) was monitored. All data were acquired and analysed with Chemstation for LC System software. The method was validated for precision linearity, selectivity, limit of detection and quantification as in [25]. Calibration curves were obtained for chlorpropham and chloroaniline quantification by spiking known concentrations of chlorpropham and chloroaniline standards into crude algal extracts and supernatant and preparing serial dilution (at least 7 points) within the range needed for the experiment. At least three fresh solutions for each concentration were used, and the mean and standard deviation was obtained. Each area value was plotted against each corresponding concentration to obtain the best-fitted line. To detect the presence of 3-chloroaniline in the biomass of *D. salina*, cultures were treated with an increasing concentration of chlorpropham (up to 1 mM) and aliquots of cultures (from 10 to 100 mL) were harvested and extracted. Then, the solvent was evaporated with the vacuum evaporator Genevac and resuspended to the compatible mobile phase.

### 4.5. Confocal Microscopy Analysis

The cell volume of *D. salina* in treated cultures was measured with an LSM880 Bruker confocal microscope. Two triplicate sets of cultures with different cell densities were set up, with the culture of lower density diluted from the one of higher density. Both cultures were treated with 20 µM chlorpropham. The laser channel was set at 488 nm, and 2D images were acquired as follows: the speed was set at 5, and the averaging number was 4. Images size was: 425.1 µM × 425.1 µM, and the pixel size was 0.42 µm. *D. salina* cells were fixed with formalin (2%) before analysis. Different images for each culture flask were acquired to have at least 50 to 100 cells per replicate flask to measure, and the volume of the cells was calculated by measuring their diameter and assuming that *D. salina* has a spherical shape.

### 4.6. Chlorpropham Removal by Washing Process

To eliminate the traces of chlorpropham surrounding the biomass of *D. salina,* samples were washed with water or the culture medium. Furthermore, the amounts of chlorpropham derived from the biomass washed with different washing cycles (0, 2, 5 and 10) or different washing solutions/harvest ratios were determined. Samples of each washing experiment were collected in triplicate from the same culture flask. The washing experiments conducted with different washing cycles were performed as follows: 10 mL culture was harvested via centrifugation at 3000× *g* for 5 min, the supernatant discharged, and the biomass pellet was resuspended completely in 10 mL washing solution (either culture medium or dH_2_O) by vortexing for 5 s; the suspension was centrifuged at 3,000× *g* for 5 min. For the experiment conducted with different harvest cultures/ washing solution volumes, the ratios were as follows: (1) 10 mL culture/10 mL washing solution; (2) 10 mL culture/20 mL washing solution; (3) 10 mL culture/40 mL washing solution.

### 4.7. Statistical Analysis

Data are presented as the mean and standard deviation of triplicates. IBM SPSS statistics 64-bit was used to perform ANOVA analysis with a significant level of *p* < 0.05 to compare the significance between data at different treatment times and between control and treatment cultures. Microsoft Excel for Office MSO 64-bit was used for graphs representations.

## Figures and Tables

**Table 1:** Amount of chlorpropham (expressed in µg) removed from the biomass of *D. salina* was washed with a different number of cycles by fresh culture medium or dH_2_O; 10 mL of culture was harvested and washed with 10 mL of washing solution.

Wash Type	Chlorpropham Removed [µg]	Loss, % Chlorpropham	Tot Intact Cell Number (×10^5^)
No wash	0	0	266.10 ± 2.90
Wash (×2)	7.446 ± 0.353	83.89	262.11± 44.5
Wash (×5)	8.681 ± 0.314	97.77	250 ± 25.70
Wash (×10)	8.848 ± 0.337	99.69	162 ± 19.50
dH_2_0 wash (×2)	8.7109 ± 0.274		

**Table 2:** Values of the amount of chlorpropham (pg/cell) with different wash solution/ harvest volumes (mL) tested.

*v*/*v* Wash Solution/Harvest (mL)	Chlorpropham Content	Chlorpropham Bound to Biomass (%) (pg/Cell)
**1/1**		
washed	0.066 ± 0.007	11.2
unwashed	0.59± 0.008	
**2/1**		
washed	0.035 ± 0.004	5.93
unwashed	0.063 ± 0.093	
**4/1**		
washed	0.015 ± 0.003	1.6
unwashed	1.10 ± 0.021	

**Table 3:** Values of retention time (min) and UV absorption λ_max_ (nm) of chlorpropham and 3-chloroaniline standards and extracts from the biomass of *D. salina*.

Property	Chlorpropham Standard	Chlorpropham Biomass	3-CA Standard	3-CA Biomass
HPLC retention time (min)	6.39 ± 0.014	6.37 ± 0.009	5.241	n.d.
UV absorption λ_max_ (nm)	238, 278	238, 278	242, 292	n.d.

## Appendix A

Figure A1 displays the change in cell volume over time for two sets of *D. salina* cultures with different cell density (high = 1.36 × 10^6^/mL and low = 0.4 × 10^6^/mL) that had been treated with 20 µM chlorpropham. Figure A2 displays the difference in cell volume of cultures treated with the herbicide for a short period of time and suspended in herbicide- free fresh medium (a and b), cultures treated for all the analysis period (24 days), (c) and not treated cultures (d).
